# Mental toughness latent profiles in endurance athletes

**DOI:** 10.1371/journal.pone.0193071

**Published:** 2018-02-23

**Authors:** Joanna S. Zeiger, Robert S. Zeiger

**Affiliations:** 1 Race Ready Coaching, Boulder, CO, United States of America; 2 Department of Allergy Kaiser Permanente Southern California Region, San Diego, CA, United States of America; 3 Department of Research and Evaluation, Kaiser Permanente Southern California Region, Pasadena, California, United States of America; Teesside University, UNITED KINGDOM

## Abstract

Mental toughness in endurance athletes, while an important factor for success, has been scarcely studied. An online survey was used to examine eight mental toughness factors in endurance athletes. The study aim was to determine mental toughness profiles via latent profile analysis in endurance athletes and whether associations exist between the latent profiles and demographics and sports characteristics. Endurance athletes >18 years of age were recruited via social media outlets (n = 1245, 53% female). Mental toughness was measured using the Sports Mental Toughness Questionnaire (SMTQ), Psychological Performance Inventory-Alternative (PPI-A), and self-esteem was measured using the Rosenberg Self-Esteem Scale (RSE). A three-class solution emerged, designated as high mental toughness (High MT), moderate mental toughness (Moderate MT) and low mental toughness (Low MT). ANOVA tests showed significant differences between all three classes on all 8 factors derived from the SMTQ, PPI-A and the RSE. There was an increased odds of being in the High MT class compared to the Low MT class for males (OR = 1.99; 95% CI, 1.39, 2.83; *P*<0.001), athletes who were over 55 compared to those who were 18–34 (OR = 2.52; 95% CI, 1.37, 4.62; *P*<0.01), high sports satisfaction (OR = 8.17; 95% CI, 5.63, 11.87; *P*<0.001), and high division placement (OR = 2.18; 95% CI, 1.46,3.26; *P*<0.001). The data showed that mental toughness latent profiles exist in endurance athletes. High MT is associated with demographics and sports characteristics. Mental toughness screening in athletes may help direct practitioners with mental skills training.

## Introduction

Understanding the psychological underpinnings of mental toughness (MT) in endurance athletes is an important issue because training physical attributes within an athlete is finite (i.e. overtraining can lead to injuries, burnout, or performance decrements) [1,2] while detecting and training weaknesses in MT have no such limitations [3,4]. Because athletes represent a high risk population for mental health problems which can impact performance and well-being [5], identifying MT factors that may be underlying such problems can guide early interventions. Indeed, higher levels of MT cross-over from success in sports to parameters of improved sleep quality [6], higher life control and interpersonal confidence [7], high levels of subjective and objective performance [8] and a healthier lifestyle [9]; it has been suggested that individuals with higher MT exhibit greater emotional control which then leads to better lifestyle choices [9]. “Specifically, individuals with higher levels of MT are less likely to believe that the demands imposed by a given situation exceed their available coping resources.”[10]

Despite the understanding that some degree of MT is necessary for effective endurance sports performance [11,12], actually defining MT has vexed researchers, with a clear classification elusive. Initially, MT was viewed in relation to one’s opponents, with more recent work focusing on “subjective or goals-directed dimensions” [13] Often a series of traits or skills (collectively recognized as MT factors) have been used to “define” MT in lieu of a proper delineation [13,14,8]. It is theorized that MT is an umbrella for multiple dimensions, inclusion of which are eight potential factors: generalized self-efficacy, buoyancy, success mindset, optimistic style, context knowledge, emotion regulation, attention regulation [8] MT has thusly been described:

MT [is] a personal capacity to produce consistently high levels of subjective (e.g., personal goals or strivings) or objective performance (e.g., sales, race time, GPA) despite everyday challenges and stressors as well as significant adversities [8]. MT can [further] be defined as a state-like psychological resource that is purposeful, flexible, and efficient in nature for the enactment and maintenance of goal-directed pursuits [13].

Components of overall MT include a combination of attributes, characteristics, and strategies[13]: goal setting, visualization, stress management, emotion control, confidence, persistence, rebounding from failure, and positive-cognition [14,15]. An effort to understand the factors that comprise MT in athletes and how these factors affect performance outcomes led to the development of multi-dimensional questionnaires [12,16,17]. Two such questionnaires are the Psychological Performance Inventory-Alternative (PPI-A) and the Sports Mental Toughness Questionnaire (SMTQ) [12,17,18]. Studies using these two MT inventories have found associations between higher MT and coping, optimism [19], hardiness, successful sports performance [20], and positive energy control [21]. The MTQ48 has also been widely used to examine six subscales of MT [9,19,22], with this questionnaire showing that MT is associated with measures of academic success [9], better problem approach coping [19,23], and improved dispositional flow [24].

“MT is positioned within a broader category of concepts that are centrally valued in their own right (e.g. self-esteem, close attachments, health, and inner peace)”[13]. MT questionnaires do not specifically measure global self-esteem; close comparisons would be confidence or self-belief, however these constructs are more akin to specific self-esteem and is different than global self-esteem [25]. “Global self-esteem does predict behavior and specific self-esteem does predict psychological well-being,” but these effects mediate each other [25]. It seems, then, that measuring global self-esteem in athletes is useful in the context of MT since self-esteem and sports are intertwined. Studies of both elite and amateur athletes use measures of global self-esteem to identify its direct and moderating/mediating effect on performance [26–28]. The Rosenberg Self-Esteem Scale (RSE) measures global self-esteem [29].

Global self-esteem impacts athletes both in their sporting life and general life. Individuals with higher levels of global self-esteem show higher levels of well-being and satisfaction with life [30,31], Self-esteem is higher in those who exercise [32,33]. Self-esteem does seem to cross over between sports and life, as self-esteem mediated the relationship between physical activity and quality of life [34]. These relationships are important, as enjoyment of sport increases participation rates which in turn increases overall happiness [35]. Overall levels of well-being and happiness in athletes are positively related to sports performance [36,37]. In addition, higher levels of self-esteem are related to lower levels of self-handicapping prior to sporting events [38], and self-handicapping has a deleterious effect on performance [38]. Athletes with high self-esteem present with more positive patterns of perfectionism, specifically these athletes showed less concerns over mistakes and fewer doubts about their actions, which in turn relate to performance gains [28]. The addition of a measure of global self-esteem can add to the total picture of an athlete’s ability to adapt in sporting settings, particularly since higher levels of self-esteem are related to lower levels of anxiety [39]. High self-esteem, in the context of performance, is related to better self-regulation; this means that when there is no alternative way to accomplish the task persistence is higher, but, when persistence is a poor strategy those with high self-esteem know when to quit [40]. The distinction between persistence and quitting is important in a sporting context where negative outcomes can occur by persisting in the face of danger.

The study of MT in athletes should extend beyond excelling in one’s chosen sport as “acquiring a mindset of mental toughness might be one way that physical activity and exercise can impact an individuals’ mental health”[41]. This sentiment underscores the notion that development of MT can be beneficial outside the sporting arena, but, the sporting arena can be the anchor for such development. As such, including several markers for MT development is imperative, because an overall MT score from a single questionnaire does not explain the whole picture, revealing only a snapshot of an athlete’s MT strengths and weaknesses, particularly for questionnaires that only measure a few MT factors.

The SMTQ measures three MT traits (confidence, constancy, control), while the PPI-A measures two traits (determination, self-belief) and two practices (visualization, positive-cognition). Using the SMTQ and PPI-A in conjunction with a measure of self-esteem (e.g. the Rosenberg Self-Esteem scale) offers a more comprehensive insight into an athlete’s MT, and through latent profile analysis, an even more complete understanding of MT within and between athletes can be derived. Indeed, many of the studies regarding mental toughness in athletes have focused on elite athletes or the psychometric properties of the measurement tools with only a few studies examining how MT is related to performance, cognitions, or behaviors [7]. Examining the MT latent profile structure in endurance athletes can help expand the knowledge base about MT, namely, whether athletes fall into MT categories in which they excel or need improvement across all of the studied factors or if there are MT factor variations (e.g. high in some factors, low in others) in endurance athletes. Creating latent classes allows for the construction of subgroups characterized by multiple dimensions, a global MT type, and how these MT types differ with respect to important outcomes of performance, psychological characteristics, and demographics.

Cluster analysis has historically been used to identify MT profiles in athletes. Affective intensity and directionality were measured in athletes. Positive affective profiles were associated with better coping [42]. A cluster analysis of MT using the Psychological Performance Inventory and the Task and Ego Orientations in Sport Questionnaire in 40 athletes revealed a three-cluster solution [21]. The clusters differed on total MT, as well as showing mean differences on MT sub-scales and there were significant differences between clusters on energy control [21].

Studies examining MT using cluster analysis have had small sample sizes, [21,43] been skewed toward males [44,45], and mostly conducted in team sports [44,45]. Latent profile analysis, a more robust method than cluster analysis, using mental toughness measures has not been conducted in endurance athletes. Furthermore, previous studies of cluster analysis did not use multiple MT measures to create a comprehensive athlete MT profile nor have previous studies examined MT profiles in relation to demographics, performance, and satisfaction. The creation of MT profiles lends itself to applications of detecting low MT athletes and using interventions to change thoughts and behaviors.

As such, we aimed to identify the number of MT classes using a latent profile analysis (LPA), discern the number of athletes within each class, and characterize the MT profile of each class.

We hypothesized that MT in endurance athletes is comprised of latent classes that can be generated by the clustering of seven mental toughness factors as measured by the SMTQ (confidence, constancy, control), PPI-A (determination, visualization, positive cognition, self-belief), and self-esteem as measured by the RSE; the validity of the MT latent profiles was tested against demographics, sports characteristics, division placement, and race satisfaction.

## Methods

### Participants

This quantitative, survey study used a convenience sample. The study was approved with waiver of written consent by Solutions IRB (http://www.solutionsirb.com). Participants were assured confidentiality. Implied consent was provided by survey completion. Participants were required to be, (1) ages 18 years or older, (2) a self-declared endurance athlete, and (3) English speaking. There were no other inclusions or exclusions. The survey was administered on SurveyGizmo (https://www.surveygizmo.com) between 29 April 2016 and 12 May 2016.

Social media and email communication were used for subject recruitment, allowing for large scale targeting of potential subjects in a relatively short time. Recruitment was researcher-initiated through social media using direct posting of the recruitment call-to-action posted on Facebook pages and dedicated to various endurance athletic sports (e.g. triathlon, swimming, ultra-running, and cycling). Postings were shared by individual athletes on their personal Facebook pages. Postings were also placed on Twitter, LinkedIn, websites dedicated to endurance sports, and emails sent directly to coaches and athletes.

### Measures

Three measurement tools were used to determine the MT latent class profiles: the Sports Mental Toughness Questionnaire (SMTQ) [17], the Psychological Performance Inventory-Alternative (PPPI-A) [12] and the Rosenberg Self-Esteem Scale (RSE) [46].

The three tools were used in combination because the statistical power to correctly identify the number of classes in the latent profile analysis is increased with a higher number of indicator variables [47,48]. Although the eight indicator variables from the three tools were significantly correlated with correlations ranging from 0.18 to 0.63, there was no evidence of multicollinearity as measured by the variance inflation factors which were all less than 2.39 in a regression model [49]. The SMTQ and PPI-A have been used in combination in other studies [15,18].

#### Mental toughness

The SMTQ is a 14-item tool that measures total MT and has three sub-scales: confidence (e.g. “I have an unshakable confidence in my ability”), control (e.g. “I am committed to the tasks I have to do”), and constancy (e.g. “I worry about performing poorly”) [17]. The responses are on a 4-point Likert scale anchored by *not at all true* and *very true*. Confirmatory factor analysis has shown excellent psychometric properties [17], and the measurement tool has been highly correlated with other MT scales such as the MTQ48 (r = 0.75) [11].

The PPI-A is a 14-item tool that is based on the original PPI [50]. Four sub-scales were identified in the PPI-A: determination (e.g. “The goals I’ve set for myself as a player keep me working hard”), self-belief (“I lose my confidence very quickly”), visualization (e.g. “Thinking in picture about my sport comes easy for me”), and positive-cognition (“I can clear interfering emotion quickly and regain my focus”). The PPI-A responses are on a 5-point Likert scale anchored by *almost always* and *almost never*. Confirmatory factor analysis indicated a good fit and high factor loadings with low standard errors (Golby et al., 2007). The PPI-A has been associated with sports performance [51].

#### Self-esteem

The RSE is a 10-item tool that measures global self-esteem with responses on a 4-point Likert scale anchored by *strongly agree* and *strongly disagree* (e.g. “On the whole, I am satisfied with myself”)[46]. Higher scores indicate higher levels of self-esteem. The RSE has shown good construct validity with high factor loadings on a single-factor model [52].

Sports characteristics

Sports characteristics were also measured: the participant’s sport (running, triathlon, swimming, cycling, and other), years involved in the sport measured as a categorical variable (1–2 years, 3–5 years, 6–10 years, more than 10 years), hours per week of training (0–5 hours, 6–10 hours, 11–15 hours, 16 or more hours), race placement in the athlete’s division (top 10, 11–20, 21 or higher), and how often the athlete was satisfied with their race results (always, often, sometimes, rarely/never).

### Procedures

Descriptive, univariate, and multivariable analyses were conducted using SPSS v23. Scores were created for the factors of the SMTQ, PPI-A, and the RSE by summing the items within the factor. For every factor, a higher score indicated a higher degree of the measured factor. Descriptive statistics, correlations, and Cronbach’s alpha were measured for each of the factors.

Latent profile analysis (LPA), a model-based cluster analysis of the mixture modeling family was used to classify related individuals [53]. The use of categorical indicator variables in a mixture model is a latent class analysis whereas continuous indicator variables, such as those used in these analyses, is a LPA [54]. The advantage of LPA versus clustering methods is the statistical assignment of an individual to a latent class. This allows for comparisons between different LPA models and the computation of a posterior probability of that individual’s membership to that latent class. An average posterior probability for each class can then be calculated with higher average posterior probabilities indicating a better fitting model [53]. Another advantage of LCA is that it is scale independent; therefore, the data does not need to be standardized [53] and the assumptions of linearity and normality of data do not need to be met [55].

A series of models with increasing number of classes, from 1 to 4, was conducted to determine the best fitting model. Akaike’s Information Criteria (AIC) and Bayesian Information Criteria (BIC) were used to compare model fit, with lower values indicating a better fit [56]. The Vuong-Lo-Mendell-Rubin Likelihood ratio test (VLMR test) was also used, which compares n classes with n– 1 classes. A significant test indicates that the n-class solution is better than the n– 1 class solution [56]. Entropy, a measurement of predictive power where 0 indicates no predictive power and 1 indicates perfect prediction, was examined [53]. Finally, the average posterior probabilities for the different class solutions were considered; a model with a good fit would have high individual probabilities to a single class. LPA was conducted in Mplus version 7 [57]. Eight variables were included in the LPA: three factors from the SMTQ, four factors from the PPI-A, and the single factor RSE. Eta squared was calculated as a measure of the effect sizes [58].

After latent class identification, ANOVA tests were conducted to compare mean scores for the eight factors between the classes and a series of chi-square tests of association examined whether class membership differed by the demographic variables (age, gender), sports characteristics (years competing in sports, hours per week of training, primary sport), and sports outcomes (placement in division, satisfaction). Multinomial logistic regression was used to examine the joint relationships between the classes and the demographics, sports characteristics, and performance variables and to test for potential interactions.

## Results

### Descriptive statistics and preliminary analyses

There was a total of 1,256 respondents, of which 11 were did not complete the entire questionnaire and were excluded from the analyses. The remaining 1,245 participants included 53% female, 23% were 18–34, 34% were 35–44, 27% were 45–54, 16% were older than 55, 84% earned a Bachelor’s degree or higher, and 90% of White ethnicity. The primary sports recorded were triathlon (54%) and running (37%), with 43% of the participants competing for 10 or more years and 45% training 6–10 hours per week. Facebook was the primary recruitment site (54%) (Table 1).

**Table 1:** Characteristics of the adult endurance athlete participants (n = 1,245).

Variable	Characteristic	n	%
**Gender**	Male	578	46.6
	Female	663	53.4
**Age**	18–34	281	22.6
	35–44	426	34.2
	45–54	339	27.2
	55+	199	16.0
**Ethnicity**	White	1,124	90.2
	Other	121	9.8
**Education**	< Bachelor's degree	195	15.7
	Bachelor's degree	475	38.2
	Advanced degree	575	46.2
**Primary sport**	Triathlon	668	53.7
	Running	455	36.5
	Cycling	52	4.2
	Swimming	48	3.9
	Other	22	1.8
**Years competing**	1–2 years	114	9.2
	3–5 years	297	23.9
	6–10 years	305	24.4
	10+ years	529	42.5
**Hours/week training**	0–5 hours	73	5.9
	6–10 hours	558	44.8
	11–15 hours	446	35.8
	16+ hours	168	13.5
**Race finish in division**	Top 10	585	47.1
	11 to 20	256	20.6
	21 or higher	402	32.3
**Study referrer**	Facebook	672	53.9
	Endurance websites	359	28.8
	Email blasts	209	16.8
	LinkedIn	5	0.4

There was a significant difference between males and females in category placement (χ^2^(2) = 12.55, *P* = 0.002), with males more often placing in the top 10 than females (49% vs. 45%). There were also significant gender differences in the number of years competing in their sport (χ^2^(2) = 38.17, *P*<0.001). Only 6.4% of males were in their sport for 1–2 years compared to 11.6% of females, and 51.6% of males participated for 10 or more years compared to 34.8% of females. There were no gender differences in the number of hours per week spent training or how often a subject was satisfied with their race performances.

Internal consistency for the factors was measured by McDonald’s omega, using JASP, which is considered a more accurate approximation of a scale’s structure than alpha [59,60]. Omega ranged from 0.60 to 0.82 (Table 2). Males had higher mean scores than females for confidence (18.9 vs. 18.1, *P*<0.001), control (11.6 vs. 10.7, *P*<0.001), self-belief (16.3 vs. 15.7, *P*<0.001) and self-esteem (32.5 vs. 31.6, *P*<0.001).

**Table 2:** Means, standard deviations, alpha, and correlations (95% CI) for study variables.

MT factor	Mean	Min	Max	SD	omega	2	3	4	5	6	7	8
**1.^±^ Confidence**	18.49	8	24	2.86	0.77	0.45* (0.40–0.49)	0.40* (0.36–0.45)	0.37* (0.32–0.42)	0.36* (0.31–0.41)	0.57* (0.53–0.61)	0.61* (0.57–0.64)	0.46* (0.42–0.50)
**2. Constancy**	14.09	6	16	1.69	0.63		0.26* (0.20–0.31)	0.52* (0.47–0.56)	0.26* (0.21–0.32)	0.40* (0.36–0.45)	0.42* (0.37–0.46)	0.32* (0.27–0.37)
**3. Control**	11.09	4	16	2.46	0.68			0.09* (0.03–0.14)	0.13* (0.07–0.18)	0.42* (0.37–0.46)	0.52* (0.48–0.56)	0.50* (0.45–0.53)
**4. Determination**	12.48	3	15	1.92	0.63				0.36* (0.31–0.40)	0.36* (0.32–0.41)	0.29* (0.24–0.34)	0.18* (0.13–0.24)
**5. Visualization**	10.53	3	15	2.86	0.82					0.42* (0.38–0.47)	0.33* (0.28–0.38)	0.17* (0.12–0.23)
**6. Positive cognition**	15.75	6	20	2.29	0.73						0.63* (0.60–0.66)	0.39* (0.34–0.43)
**7. Self-belief**	16.00	7	20	2.56	0.82							0.55* (0.51–0.59)
**8. Self-esteem**	33.41	15	40	4.96	0.89							

^±^numbers refer to MT factor;

*P<0.01

### Latent profile analysis

The four LPA model comparisons indicated that a three-class solution was the best fit to the data (Table 3). The four-class model had the lowest AIC and BIC values, however, the VLMR test was not significant, indicating that four classes were not better than three classes. The three-class solution had lower AIC and BIC values than the two-class solution, a significant VLMR test, and an entropy value of 0.80. In the three-class solution, the mean posterior probabilities ranged from 90% to 92%.

**Table 3:** Latent profile comparisons with fit statistics and average class probabilities for most likely class membership by latent profile class numbers.

	**1 Class**	**2 Class**	**3 Class**	**4 Class**
AIC	47,115.9	44,899.0	44274.6	44069.1
BIC	47,197.9	45,027.2	44448.9	44289.6
SSA_BIC	47,147.1	44,947.8	44340.9	44153.0
Entropy	N/A	0.82	0.80	0.80
VLMR test	N/A	-23,542.0	-22,424.5	-22.103.3
VLMR p-value	N/A	0.00	0.00	0.16
**Estimated posterior probability**	**Average class probabilities for class membership**			
Two-class model				
1, n = 459.6, 36.9%	0.94	0.06		
2, n = 785.4, 63.1%	0.04	0.96		
Three-class model				
1, n = 236.5, 19.0%	0.92	0.08	0.00	
2, n = 575.5, 46.2%	0.04	0.90	0.06	
3, n = 433.0, 34.8%	0.00	0.09	0.91	
Four-class model				
1, n = 183.3, 14.7%	0.89	0.06	0.05	0.00
2, n = 527.7, 42.4%	0.02	0.88	0.03	0.07
3, n = 116.8, 9.4%	0.08	0.11	0.81	0.00
4, n = 417.2, 33.5%	0.00	0.09	0.00	0.91

Note. AIC = Akaike Information Criterion; BIC = Bayesian Information Criterion; SSA-BIC = Sample-Size-Adjusted BIC; VLMR = Vuong-Lo-Mendell-Rubin Likelihood ratio test compares n with n– 1 classes. A significant test indicates that the n-class solution is better than the n– 1 class solution; Entropy = measure of how well a model predicts class membership, ranging from 0 (no predictive power) to 1 (perfect prediction).

The three classes were designated relative to MT as “High MT” (n = 433, 34.9%), “Moderate MT” (n = 579, 46.5%) and “Low MT” (n = 233, 18.7%) based on the factor means for each class, which were significantly different for all eight factors between all three classes tested with ANOVA and a post-hoc Bonferonni test (Table 4). A minimum effect size greater than 0.2 is considered the lower level for practical significance, with 0.5 indicating a moderate effect and 0.8 a strong effect [61]. The smallest effect sizes were 0.18 for visualization and 0.19 for determination. The highest effect sizes were 0.65 for self-belief, 0.52 for positive cognition and confidence.).

**Table 4:** Means and standard deviations by classes for mental toughness factors.

	Class								
MT Factors	High MT		Moderate MT		Low MT		F*	*P*	Effect size
	(n = 433, 34.9%)		(n = 579, 46.5%)		(n = 233, 18.7%)				
	Mean	SD	Mean	SD	Mean	SD			
**Confidence**	20.86	1.91	18.12	1.91	15.00	2.24	683.82	<0.001	0.52
**Constancy**	15.12	1.04	13.99	1.46	12.45	1.84	272.94	<0.001	0.31
**Control**	12.73	2.03	10.80	2.06	8.78	1.95	298.22	<0.001	0.32
**Determination**	13.42	1.45	12.34	1.75	11.04	2.10	147.46	<0.001	0.19
**Visualization**	11.94	2.61	10.29	2.52	8.49	2.73	138.64	<0.001	0.18
**Positive cognition**	17.73	1.45	15.33	1.56	13.13	1.91	671.11	<0.001	0.52
**Self-belief**	18.35	1.30	15.65	1.54	12.49	1.8	1160.97	<0.001	0.65
**Self-esteem**	36.68	3.37	33.14	4.01	27.99	4.58	374.58	<0.001	0.38

*Post-hoc Bonferroni tests showed significant differences between all factors for all classes

### Univariate analyses

Males (χ^2^(2) = 13.43, *P* = 0.001), older age groups (χ^2^(6) = 16.03, *P* = 0.01), athletes who competed for more years (χ^2^(6) = 30.35, *P*<0.001) and participants who trained more hours (χ^2^(6) = 14.80, *P* = 0.02) were in the High MT cluster more often than females, younger age groups, newer athletes, and those who trained fewer hours, respectively (Table 4). Satisfaction with performance (χ^2^(6) = 187.35, *P*<0.001) and a top 10 category placement (χ^2^(4) = 35.07, *P*<0.001) showed a higher prevalence of being in the High MT cluster compared to lower satisfaction and higher category placement, respectively (Table 5). There were no differences in class membership by education or primary sport.

**Table 5:** Associations between demographics and sports characteristics and MT classes.

		High MT		Moderate MT		Low MT		
Variable	Characteristic	n	%	n	%	n	%	Test
**Gender**	Male	196	33.8	276	47.6	108	18.6	χ^2^(2) = 13.43, *P =* 0.001
	Female	182	27.4	316	47.6	166	25.0	
**Age**	18–34	70	24.9	145	51.6	66	23.5	χ^2^(6) = 16.03, *P* = 0.01
	35–44	119	27.9	205	48.1	102	23.9	
	45–54	112	32.9	152	44.7	76	22.4	
	55+	77	38.3	92	45.8	32	15.9	
**Education**	< Bachelor's degree	67	34.4	90	46.2	38	19.5	χ^2^(4) = 353, *P* = 0.47
	Bachelor's degree	144	30.3	225	47.3	107	22.5	
	Advanced degree	167	28.9	279	48.4	131	22.7	
**Years competing**	1–2 years	29	25.4	48	42.1	37	32.5	χ^2^(6) = 30.35, *P*<0.001
	3–5 years	69	23.2	152	51.0	77	25.8	
	6–10 years	92	30.2	143	46.9	70	23.0	
	10+ year	188	35.4	251	47.3	92	17.3	
**Hours/week training**	0–5 hours	24	32.9	28	38.4	21	28.8	χ^2^(6) = 14.80, *P* = 0.02
	6–10 hours	145	25.9	277	49.6	137	24.5	
	11–15 hours	144	32.2	216	48.3	87	19.5	
	16+ hours	65	38.5	73	43.2	31	18.3	
**Satisfaction with performance**	Always	53	68.8	20	26.0	4	5.2	χ^2^(6) = 187.35, *P*<0.001
	Often	247	35.1	357	50.7	100	14.2	
	Sometimes	71	17.4	193	47.4	143	35.1	
	Rarely/Never	7	11.7	24	40.0	29	48.3	
**Place in category**	Top 10	208	35.4	286	48.7	93	15.8	χ^2^(4) = 35.07, *P*<0.001
	11–20	72	28.0	129	50.2	56	21.8	
	21 or higher	96	23.9	179	44.5	127	31.6	
**Primary sport**	Triathlon	205	30.6	311	46.5	153	22.9	χ^2^(8) = 9.47, *P* = 0.30
	Running	140	30.6	221	48.4	96	21.0	
	Swimming	14	29.2	23	47.9	11	22.9	
	Cycling	10	19.2	29	55.8	13	25.0	
	Other	9	40.9	10	45.5	3	13.6	

### Multivariable analyses

**Fig 1:**
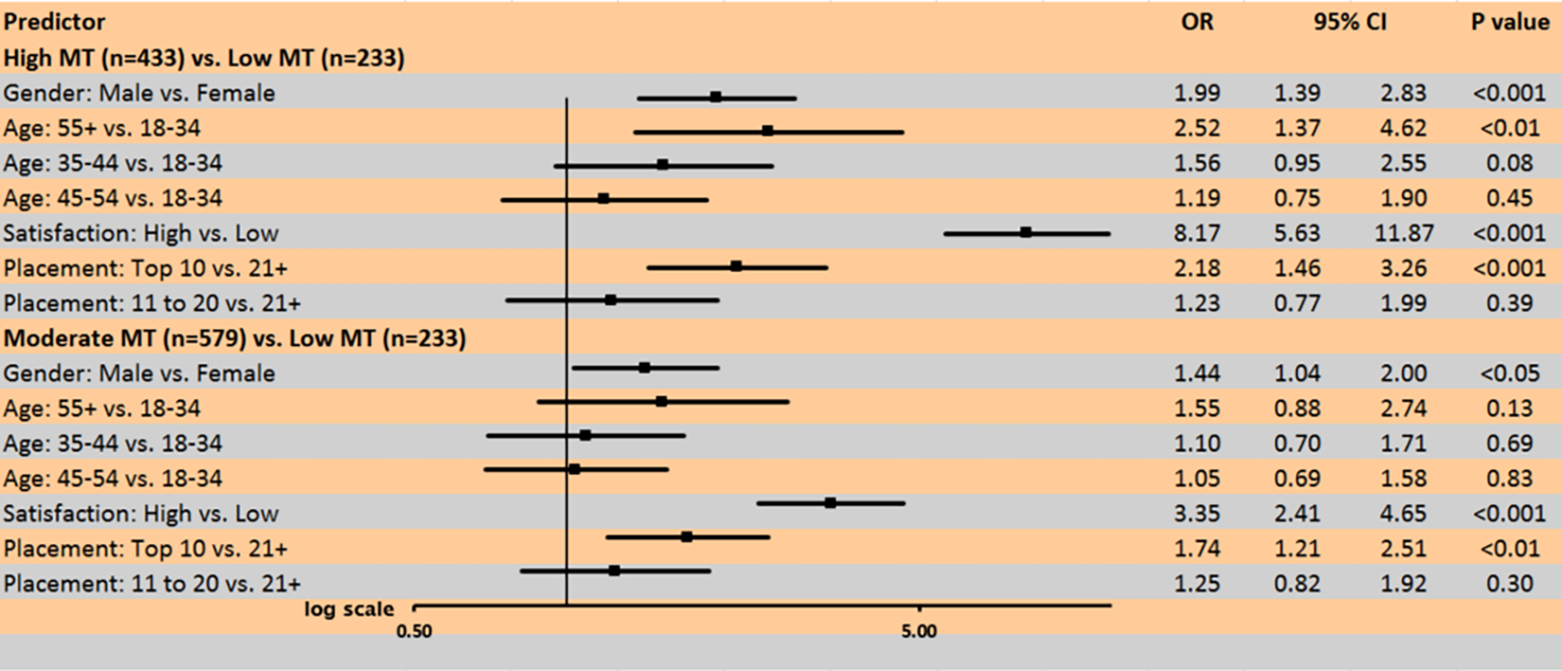
Multivariable analyses of associations between mental toughness (MT) latent class membership and sports and demographic variables. High MT (n = 433), Moderate MT (n = 579), Low MT (n = 233). High satisfaction = always or often; low satisfaction = sometimes, rarely or never.

The results from the most parsimonious multinomial logistic regression model are shown in Fig 1. The Low MT cluster served as the reference group. Satisfaction was dichotomized into High (Always and Often) and Low (Sometimes, Rarely, or Never). There were no significant interaction effects. Males, athletes older than 55 years, High satisfaction, and a top 10 division placement increased the odds of being in the High MT cluster when compared to females (OR 1.99, 95% CI 1.39–2.83, *P*<0.001), 18–34 year olds (OR 2.52, 95% CI 1.37–4.62, *P*<0.05), Low satisfaction (OR 8.17, 95% CI 5.63–9.11.87, *P*<0.001) and placing 21 or higher (OR 2.18, 95% CI, 1.46–3.26, *P*<0.001). Membership in the Moderate MT cluster compared to the Low MT cluster was significantly associated with gender (males vs. females: OR 1.44, 95% CI, 1.04–2.00, *P*<0.05), satisfaction (High vs. Low: OR 3.35, 95% CI 2.41–4.65, *P*<0.001) and placement in one’s division (top 10 vs. 21 or higher: OR 1.74, 95% CI, 1.21–2.51, *P*<0.001).

## Conclusions

We examined whether there are identifiable MT latent classes in athletes who participate in endurance sports and if the MT profiles are associated with demographics, sports characteristics, satisfaction with race results, and division placement. Eight factors from three measurement tools were used to improve both statistical power and clinical utility. Three classes emerged that corresponded to High, Medium, and Low MT based on mean scores for the eight measured factors. Multivariable analysis indicated that males, older athletes, high-ranking division placement, and high levels of race satisfaction all predicted membership into the High MT class as compared to the Low MT class.

Enrollment of a large sample size of athletes who participated in endurance sports with an equitable male to female ratio made this study novel. Additionally, until now, it was unknown whether the MT profiles of endurance athletes mirror that of team sport athletes.

These results corroborate the findings which found three MT profiles in adolescent cricket players corresponding to low, medium, and high MT [45]. The inventory in that study was specific to cricket, however, the five sub-scales of self-belief, affective intelligence, resilience, attentional control, and desire overlapped with those used in the current study. Additionally, in that study, the low and high clusters showed low and high levels across all five measured traits, respectively, a finding also observed in the current study. A study of older adolescent Australian football players indicated a two cluster profile, high and low; the effect sizes for the MT factors in that study were similar to those observed in these analyses [44].

The mean scores for the measured traits by MT cluster in this study fit into the competitive standards created by a study of the SMTQ and the PPI-A in 455 athletes (76% male) from 19 different sports (10% endurance sports) competing at the elite and sub-elite level [18]. Sheard concluded that the PPI-A and SMTQ used together represent a valid and reliable means of measuring MT and these measures can be used to evaluate an athlete’s MT over time, particularly if an intervention of mental skills training has been implemented [18].

Inclusion of females was informative in that there were significant gender differences in class membership. Females were less likely to be in the High MT class, and showed lower levels of the specific traits of confidence, self-esteem, self-belief, and control. Studies have shown lower levels of self-esteem in females [34], and confidence and control as measured by the SMTQ was lower in females than males [17]. These results indicate that the PPI-A, SMTQ, and RSE used together adequately distinguish sub-populations of athletes who need targeted mental skills training.

The relationship between MT and satisfaction is one of particular interest and fits in with the Basic Need Theory, which postulates that “humans function and develop effectively as a consequence of the social environment and its potential for basic need satisfaction”[62]. Sports satisfaction has been related to physical well-being [63], pre- and post-practice well-being [64], athletic performance [65], and sports vitality [62].

In addition, long term adherence to sports improves with increased levels of satisfaction [66,67]. A recommendation for a new model of understanding and implementing sports participation focuses on creating higher levels of sports satisfaction through skills development rather than the current paradigm of increasing motivation and changing behaviors [68]; MT factors are such skills that can be developed over time to increase sports satisfaction.

We propose that the formation of MT classes, as determined in this study, broadens the scope of knowledge about MT profiles in endurance athletes by contextualizing the individual MT factors. Further studies of MT using these measures in endurance athletes will be needed to replicate these results. The field of MT has been hindered by the complexity of the concept. Gucciardi postulated that MT can be defined “as a state-like psychological resource that is purposeful, flexible, and efficient in nature for the enactment and maintenance of goal-directed pursuits.”[13] Within this framework, understanding that MT is flexible, exemplifies that MT can be trained. Therefore, using techniques to identify weaknesses, such as easily administered questionnaires used in this study, is important for coaches and practitioners. Additionally, this study suggests that endurance athletes’ MT is consistent across dimensions, so identifying athletes who are at the lower end can potentially improve not only their proficiency in sports, but also improve their general well-being.

Self-belief had the highest effect size. A group of elite level international athletes ranked self-belief as the most important dimension of MT [14,69], with self-belief having four attributes. “The attributes in this subcomponent relate to factors that contribute to performers’ unshakable belief through their awareness and inner arrogance and how this belief results in performers’ reaching their true potential, despite obstacles and barriers that people or organizations put in their path.[69]”

This study had a few limitations. It is unknown whether the participants were answering the questions honestly; however, the anonymity of the questionnaire increased the likelihood of truthful responses. The generalizability of this convenience sample drawn from social media outlets is unknown. However, comparisons to the latest statistics from the governing body of triathlon (USA Triathlon) and the USA Running State of the Sport trends show that the participant demographics in this sample roughly match the overall populations of running and triathlon, the two sports most largely represented in this sample [70,71]. Even though the sample demographics reflect those of the greater population of triathletes and runners, the participants are self-selected, therefore the MT classes may not be representative of endurance athletes in general.

In summary, using the RSE and two validated MT questionnaires, the PPI-A and SMTQ, in a large cross-sectional sample of adult endurance athletes, we identified three latent profiles that corresponded to High, Medium, and Low MT. The MT profiles are associated with satisfaction with race results, gender, age, and race placement. A scoring algorithm from a discriminant analysis will be developed based on the latent profiles results which will allow clinicians and coaches to screen MT in athletes using the RSE, PPI-A, and SMTQ.

## Abbreviations

MT: mental toughness
SMTQ: Sports Mental Toughness Questionnaire
PPI-A: Psychological Performance Inventory-Alternative
RSE: Rosenberg Self-Esteem Scale
